# The effectiveness of video interaction guidance in parents of premature infants: A multicenter randomised controlled trial

**DOI:** 10.1186/1471-2431-12-76

**Published:** 2012-06-18

**Authors:** Anneke Tooten, Hannah N Hoffenkamp, Ruby AS Hall, Frans Willem Winkel, Marij Eliëns, Ad JJM Vingerhoets, Hedwig JA van Bakel

**Affiliations:** 1 International Victimology Institute Tilburg, Tilburg University, Tilburg, the Netherlands; 2 Centre for Psychotrauma, Reinier van Arkel group, ’s, Hertogenbosch, the Netherlands; 3 Association Interactionguidance and Treatment, Foundation and Combination Youth care, Eindhoven, the Netherlands; 4 Department Developmental Psychology, Tilburg University, Tilburg, the Netherlands; 5 Dimence Institute, Centre of Infant Mental Health, Deventer, the Netherlands

## Abstract

**Background:**

Studies have consistently found a high incidence of neonatal medical problems, premature births and low birth weights in abused and neglected children. One of the explanations proposed for the relation between neonatal problems and adverse parenting is a possible delay or disturbance in the bonding process between the parent and infant. This hypothesis suggests that due to neonatal problems, the development of an affectionate bond between the parent and the infant is impeded. The disruption of an optimal parent-infant bond -on its turn- may predispose to distorted parent-infant interactions and thus facilitate abusive or neglectful behaviours. Video Interaction Guidance (VIG) is expected to promote the bond between parents and newborns and is expected to diminish non-optimal parenting behaviour.

**Methods/design:**

This study is a multi-center randomised controlled trial to evaluate the effectiveness of Video Interaction Guidance in parents of premature infants. In this study 210 newborn infants with their parents will be included: n = 70 healthy term infants (>37 weeks GA), n = 70 moderate term infants (32–37 weeks GA) which are recruited from maternity wards of 6 general hospitals and n = 70 extremely preterm infants or very low birth weight infants (<32 weeks GA) recruited by the NICU of 2 specialized hospitals. The participating families will be divided into 3 groups: a reference group (*i.e.* full term infants and their parents, receiving care as usual), a control group (*i.e.* premature infants and their parents, receiving care as usual) and an intervention group (*i.e.* premature infants and their parents, receiving VIG). The data will be collected during the first six months after birth using observations of parent-infant interactions, questionnaires and semi-structured interviews. Primary outcomes are the quality of parental bonding and parent-infant interactive behaviour. Parental secondary outcomes are (posttraumatic) stress symptoms, depression, anxiety and feelings of anger and hostility. Infant secondary outcomes are behavioral aspects such as crying, eating, and sleeping.

**Discussion:**

This is the first prospective study to empirically evaluate the effect of VIG in parents of premature infants. Family recruitment is expected to be completed in January 2012. First results should be available by 2012.

**Trail registration number:**

NTR3423

## Background

Each year, 2% to 9% of the newborns require specialised care in neonatal intensive care units (NICU). The majority are premature infants (born before 37 weeks of gestational age) who weigh less than 2500 g at birth. Modern medical technology has forced back the frontiers of viability so that a growing number of babies, even as young as 23 to 24 weeks gestation with weights as low as 500 gram, are currently surviving [1]. With the improved survival chance of preterm infants, there is a growing concern for their developmental outcome and future quality of life.

Studies have consistently found a high incidence of abuse among children with a history of neonatal medical problems, premature birth and low birth weight [2,3]. Infants experiencing poorer fetal growth or preterm birth are at increased risk of physical, emotional, or abuse or neglect independent of maternal age and socioeconomic status [4]. One of the explanations proposed for the relation between neonatal problems and non-optimal parenting is a delay or disturbance in parent-infant bonding. This hypothesis suggests that due to neonatal problems, the development of an affectionate bond between the parent and the infant is impeded [5]. The disruption of an optimal parent-infant bond -in its turn- may pre-dispose distorted parent-infant interactions and thus facilitate abusive or neglectful behaviours. However, this hypothesis has not been tested empirically in a prospective study.

### Bonding & attachment

Parental bonding and attachment are two interrelated concepts. “*Bonding*” can be described as: “the establishment of an emotional connection of the parent to the infant” [2]. This bond is not assumed to be bidirectional *per se*; it is more seen as unidirectional, from the parent to the infant. The process of forming a bond with a baby begins during pregnancy and develops further after birth. Forming such a bond is fundamental for the development of the baby [6]. The process of bonding, in its turn, sets the stage for the evolvement of attachment, which develops later in childhood [7], and which can be described as: ‘the capacity to form selective, enduring and mutual relationships” [8-10].

Premature birth may impede or disturb parental bonding and the later relationship between parent and child. The process of bonding from the parent to their infant may be compromised due to several causes. [11]. Early separation attributable to the infant’s bio-medical complications, invasive medical treatments, as well as the anticipated loss of the baby may result in physical and emotional distance between parents and their preterm newborn [12]. These circumstances can be so emotional, frightening and overwhelming for parents that they turn away from their baby. Alternatively, these feelings may push them to overstimulate the baby in a desperate search for a reassuring response from the infant. At the same time, parental negative feelings (*e.g.* confusion, detachment, fear) may impede the establishment of a well-balanced parent-infant relationship and can be the source of parent-infant attachment difficulties [13,14].

### Psychological stress responses

The first years after birth are a unique emotional experience for most parents, also when the infant is born at term and in good health. [15]. However, parents of preterm infants face many specific problems engendered by timing of birth, a prolonged hospital stay, distinctive patterns of behaviour, and development in the infant’s early years. Parents’ expectations for a normal delivery and giving birth to a healthy infant are violated, and they must come to terms with disappointment and possible loss as well as fears for their infant’s health and future [16,17]. Parents of premature infants nurture under stressful, hectic and worrying circumstances. This stressful aspect of preterm birth and its psychological impact on parents have long been acknowledged [18].

The stressful nature of the Neonatal Intensive Care Unit (NICU) environment for parents is also well documented. The physical environment is a major source of stress for parents, with bright lights, noisy life support and monitoring equipment, and chemical scents. Furthermore, viewing their ill infant connected to equipment by tubes and wires and surrounded by medical personal can be very disturbing. However, the greatest source of stress experienced by these parents is often the loss of their expected and desired parental role. Parents often report feelings of disappointment and frustration because they cannot perform their normal parenting task (*e.g.* feeding) as they had expected. Moreover, they also may feel extreme distress and helplessness about not being able to protect their infant from harm [19].

Parents’ emotional reactions to the NICU experience can vary from disappointment, guilt, sadness, depression, hostility, anger, fear, anxiety, grief, helplessness to a sense of failure and loss of self-esteem [20]. After birth of a premature infant, high levels of depression and anxiety are common for both parents [21,22]. One month after birth, mothers of premature infants have been found to be at greater risk of psychological stress than mothers of full-term infants [23], with 10% of mothers of premature infants in one study experiencing severe symptoms of psychological distress neonatally and one third experiencing clinically levels of depression and anxiety [24].

Only recently a few studies have examined preterm birth and parents’ experiences from a trauma perspective. Studies indicate that parents of preterm infants report a high incidence of PTSD reactions, still lasting 1 year after the infant’s birth [25-28]. Feelings of depression, anxiety and post traumatic stress may negatively interfere with parent-infant interaction [29].

### Parent - infant interaction

The quality of the parent-infant interaction is an important mediating factor between perinatal risk and later infant competencies. Important characteristics of parent-infant interaction are sensitive and responsive interactional behaviour, which -in its turn- fosters optimal infant cognitive and social development [24,30-33]. ‘Parental sensitivity’ can be described as: the ability to perceive infant’s signals accurately, and ‘parental responsiveness’ as the ability to respond to these signals promptly and appropriately [34]. Well-timed parent-infant interaction attuned to infants’ cues, helps to regulate infants’ physiological (*e.g.* heart rate, respiration and body temperature), behavioural, social and emotional responses (*e.g.* distress) [35].

The birth of a premature infant and its hospitalization interrupt the expected development of interactive skills for both the parents and the infant. First of all, parents cannot hold and nurture their baby frequently or spontaneously. In addition, parents are dependent on the nursing staff to support them. Adding to the stressful situation, the distinctive physical appearance and behavioural characteristics of premature infants may also impede the development of positive parent-infant relationships [36]. 

Research has shown that the appearance of preterm infants is judged as less attractive than full term infants and their behaviour is observed as less alert, less attentive, less active and less responsive than that of full-term infants. Furthermore, preterm infants engage in fewer broad smiles, are relatively fussy and irritable, are more difficult to soothe, show more mixed behavioural cues, show more sensory-defensive behaviours and are described as more temperamentally difficult than term peers. Moreover, preterm infants diverge in the way they cry; babies who have experienced stressful medical conditions differ acoustically from healthy infants, namely the sound of their crying is perceived as more aversive and physiologically arousing to adults than those of full-term infants [37-47].

Several studies have examined the interaction styles of parents, in particular mothers, of preterm infants during the neonatal period [1,37-39,48-50]. However the findings until now are still inconclusive. Some preterm mothers are focused in their interaction towards stimulation, while others show more affective withdrawal. A possible mediating factor in interaction style is the presence of post traumatic stress in parents. Mothers of preterm infants with post traumatic stress symptoms were more likely to have “controlling” dyadic patterns of interaction and to show distorted infant representations. Preterm infants of these “controlling” dyads have significantly less positive outcomes compared to full-term infants. They display more behavioural problems (particularly eating problems) and have lower developmental social skills.

The (over)stimulating approach of preterm mothers has been a point of discussion, considered by some authors as an adaptive and compensatory reaction to the specific difficulties presented by the preterm infant’s immaturity, and seen by others as intrusive and controlling behaviour, unfavourable to the preterm infant’s outcome [1,38,51]. These distinct findings can partially be explained by major advances in neonatology over the last 20 years, to greater parental attendance and participation in the infant’s care in the NICU, as well as to the improved emotional support given to the parents during the neonatal period. However, since smaller and more immature preterm infants are currently surviving, with longer hospitalisations; parent-infant interactions and parent-infant relationships are still at risk [52].

The barriers to parenting experienced in the NICU and parents’ psychological stress responses after delivery of premature infants, may negatively influence the parent-infant relationship and the infants’ long-term developmental outcome. The postponement of parenting and the emotional and psychological stress, may cause parents not being able to emotionally connect to their infant at time of discharge, and may contribute to greater parenting risk and child vulnerability [24,53,54]. These findings highlight the importance of therapeutic interventions during hospitalisation in the NICU aiming at improvement of parent-infant interactions and early parental- therapeutic support focusing on the psychological impact after premature birth. [15].

### Prevention programmes

In general, early individualized family based interventions during neonatal hospitalisation and the transition to home, have been shown to reduce parental stress and depression, increase parental self-esteem, and improve positive early parent-preterm infant interactions [55]. During hospitalisation parental self-confidence has to be reinforced repetitively and evaluated before discharge because insecure parents at discharge are more likely to have problems with their infants at home, which may lead to persistent parent-infant relationship problems [15].

Parents with infants at risk (*e.g.*, ill or premature infants) may need additional support to develop well-balanced positive relationships. Parents of premature infants often experience ambivalent or negative emotions toward their infants and/or about themselves during hospitalisation and after discharge. Half of the mothers of very preterm infants felt that they had to cope with negative feelings when first seeing their infant and 65% had negative or ambivalent feelings in the first weeks at home after hospital discharge. These results suggest that these mothers may require support in coping with negative feelings concerning these early experiences. Parental support could start immediately after birth to promote initial moments of positive interaction between parent and infant [29].

Preventive post-hospital discharge interventions focused at preterm infants and their parents may improve social and emotional developmental outcomes [56-58]. Research has shown that preventive trauma intervention for mothers resulted in significantly less traumatic impact at discharge, although without intervention 77% of preterm mothers showed significant psychological trauma 1 month after birth and 49% 1 year later [27]. Joined observations of the infant’s social cues guided by mental health practitioners, help parents to better understand and attune to their infants individual characteristics and the premature infant’s salient limitations in their social interaction. The process of joint observation stimulates parental attention and preoccupation with the baby, parental competence in reading the infant’s cues and responding sensitively and responsively to the infant’s behaviour. It further provides them with the opportunity to nurture the infant while experiencing an affective and positive experience, which reinforces the parent-infant bonding process [59]. It enables parents to overcome often frightening, traumatic images of their infant and it prevents the beginning of a negative vicious circle of pessimistic emotions, which could threaten the development of a harmonious parentinfant relationship [15].

### Video interaction guidance (VIG)

Video Interaction Guidance (VIG) is a method for nurses and pedagogic workers in the clinical (hospital) setting to guide and support the attunement and positive contact between parent and infant during the hospital stay [60]. VIG uses edited video feedback to help parents identify their strengths and to achieve desired goals. Key elements of the method are adoption of a collaborative and empowering approach to the parent and to offer a framework of theoretically derived communication/contact principles to analyze interactions. Edited film elements are used to provide feedback of “positive exceptions” and, through discussion of these self-modeling examples, to facilitate reflection and develop parental self-efficacy. VIG interventions consist of approximately 3–5 sessions.

International studies have shown similar short-term interaction guidance interventions to be effective in increasing positive caregiver behaviour. After two video feedback sessions a significant reduction in the degree of negativity of parental attributions towards their child was found [61] and a significant decrease of disrupted behaviour following two sessions was observed [62].

## Methods/design

### Aims and hypotheses

The primary aim of this study is to evaluate the effectiveness of Video Interaction Guidance in parents of premature infants. We hypothesise that VIG enhances parental bonding after premature childbirth and prevents adverse parent-infant interaction.

The secondary aim is to further elucidate the bonding process between parents and their preterm infants. We hypothesise that the bonding process in parents with premature infants is delayed compared to parents with full term infants.

### Research design

**Figure 1:**
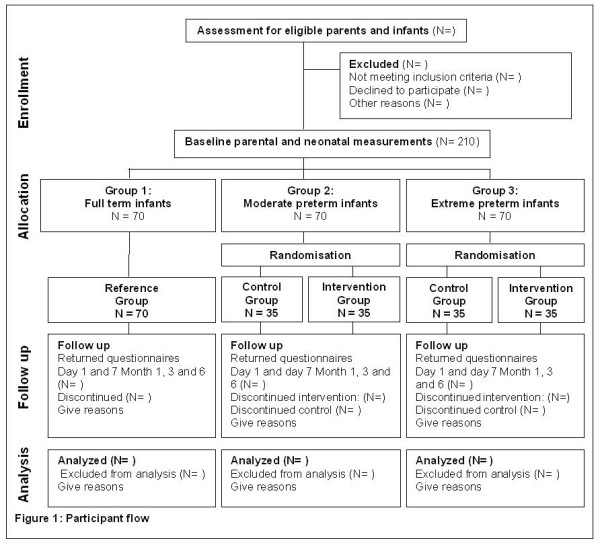
Participant flow.

This is a multi-center randomised controlled trial (RCT) to examine the effectiveness of Video Interaction Guidance. The RCT will be conducted in the south of the Netherlands in 8 hospitals. In total 210 newborn infants with their parents will be included in this study. Healthy term infants (>37 weeks GA, n = 70) and their parents and moderate term infants (32–37 weeks GA, n = 70) and their parents will be recruited from maternity wards of 6 general hospitals. Extremely preterm infants or very low birth weight infants (<32 weeks GA, n = 70) and their parents will be recruited by the NICU of 2 specialized hospitals The participant flow is displayed in Figure 1.

### Recruitment

Parents will be recruited within 24 hours after delivery. They will be asked to participate in this study by a nurse/gynaecologist or pediatrician. The parents will be well informed through an information brochure and a letter about the aims, the implications of the study and the intervention in general (*i.e.* the amount of time and the home visits). When both parents agree to participate, they have to sign an informed consent form.

### Randomisation

Once informed consent is obtained, the parents and newborn infants will be divided into 3 groups: a reference group, a control group or an intervention group. The reference group contains all healthy term infants (>37 weeks), whereas the moderate (32–37 weeks GA) and the extreme preterm infants (<32 weeks GA) will be randomly allocated to either the control or the intervention group. Randomisation will be arranged by using computer-generated random numbers. The allocation of the control or intervention group is performed by using sealed envelopes.

### Trial procedures

All parents and their newborn infants will receive standardized hospital care after delivery (care as usual). Furthermore, depending on the group into which parents are divided, several interaction moments between the parents and their infant will be recorded on video during the first week after delivery. Parents from the reference group will be recorded on video once within the first 24 hours after delivery. Parents allocated to the control group, will be recorded on video twice within the first week, respectively at day 1 and 7. Parents allocated to the intervention group, will be recorded on video three times within the first week, respectively at day 1, 4 and 7. The parents from the intervention group will receive feedback on these recordings from the VIG- nurses, whereas parents from the reference and the control group will not receive feedback.

### Measures

The data includes observations of parent-infant interactions, questionnaires, and semi-structured interviews. Data collection was started in September 2009 and will be completed in December 2011. The measurements from both parents will be conducted at day 1, 3 and 7 and month 1, 3 and 6. The time schedule of the measurements can be found in Table 1.

**Table 1:** Time schedule measurements

**Measures**	**Time point**					
	**Day 1**	**Day 3**	**Day 7**	**Month 1**	**Month 3**	**Month 6**
**Parental variables:**						
Parent-Infant Interaction	•	•	•	•		•
Parental bonding	•	•		•	•	
Stress	•			•	•	•
Depression				•		•
Anxiety				•		•
Anger				•		•
Personality					•	
Ego-resilience					•	
Support					•	
**Neonatal variables**						
Medical information	•	•	•	•	•	•
Behaviour	•	•	•	•	•	•
Temperament					•	

### Primary outcomes

Parental bonding and parent-infant interactive behaviour are the primary outcomes of this study. To assess parental bonding the following tools will be used: the Pictorial Representation of Attachment Measure (PRAM) [63], the My baby and I questionnaire [64], the Postpartum Bonding Questionnaire (PBQ) [65], the Yale Inventory of Parental Thoughts and Actions questionnaire (YIPTA) [66,67], the Mother And Baby Scale (MABS) [68] and the Parental Bonding Instrument (PBI) [69]. Interviews which are used are: the Clinical Interview for Parents of high-risk infants (CLIP) [70] and the Working model of the Child Interview (WMCI) [71]. Parent-infant interactive behaviour will be rated from videotapes which are recorded during interaction moments. To assess parent-infant interaction the Emotional Availability Scales (EAS) [72] will be used. The EAS contains the following five subscales: sensitivity, structuring, intrusiveness, responsivity and involvement, measured on a 5 or 9 point Likert-scale.

### Secondary outcomes

#### Parental secondary outcomes are

Stress, will be measured by the Traumatic Event Scale (TES) [73], the Parental Stress Scale Neonatal Intensive Care Unit (PSS-NICU) [74], the Perinatal PTSD Questionnaire (PPQ) [75] and the Perceived Stress Scale (PSS) [76].

Depression, will be measured by the Edinburg Postnatal Depression Scale (EPDS) [77].

Anxiety: will be measured by the State-Trait Anxiety Inventory (STAI) [78].

Anger: will be measured by the State-Trait Anger Expression Inventory (STAXI) [79].

Satisfaction with hospital care and intervention: will be measured by the Nurse Parent Support Tool (NPST) [80] and supplementary contentment questions about hospital care and if received: the intervention VIG.

#### Infant secondary outcomes are

Behaviour of the infant: will be measured by subscales of the Infant Behaviour Questionnaire Revised (IBQ-R) [81] and the Ages and Stages Questionnaire: Social-Emotional (ASQ- SE) [82].

### Confounders

#### Parental confounders are

Various mood states: will be measured by the UWIST Mood Adjective Checklist (UMACL) [83], the I Feel Pictures [84] and supplementary questions about crying behaviour of the parents.

Coping: will be measured by the Ego Resilience scale (ER89) [85], the Perceived Maternal Parenting Self-Efficacy questionnaire (PMP S-E) [86], the Coping Inventory Stressful Situations questionnaire (CISS) [87] the Parenting Sense of Competence Scale (PSOC) [88] and the Soothing Methods Questionnaire [89].

Personality: will be measured by the Quick Big Five (QBF) [90].

Support: will be measured by the Family Assessment Measure III, subscale: Spousal Support Scale (FAM III) [91] and the Relationship Questionnaire (RQ) [92].

Background variables: additional (personal and medical) information.

#### Infant confounders are

Temperament: will be measured by the Infant Characteristics Questionnaire (ICQ) [93].

Background variables: additional (medical) information

### Long-term follow-up

In the Netherlands, with approximately 14.000 preterm births per year and an alarming number of young children victimized by maltreatment and neglect [94] more insight into the process of parenting and potential risk or protective factors is badly needed [2,4,5]. The purpose of the follow-up study is to increase understanding of the process of parenting a premature infant and the determinants of positive and negative parental and infant outcomes later in life. The primary aim of the follow-up study is to gain more insight into the process of parental bonding, attachment and parent-infant interactive behaviour after (premature) childbirth, in relation to the infant’s development at the age of two. Controlling for gestational age, it is expected that there will be a difference in social-emotional, behavioral and cognitive development between preterm infants and full term infants, in favor of the latter group. The secondary aim of the follow-up study is to examine the long term effects of a VIG, whereas it is expected that parent-infant dyads who received VIG have a better quality of attachment than dyads that did not receive the intervention. The final aim of the study is to assess the risk of abuse or neglect. Based upon literature the risk of infant maltreatment and neglect is expected to be higher in parents of preterm infants compared to parents of full term infants.

All families (N = 210) participating in the first part of the study will be invited to take part in the follow-up study. Parents will receive a letter containing information about the follow-up study, which will be introduced as study of attachment and development of premature infants. A week after having received the letter, parents will be called and will be asked if they affirm to participate. Measurements will consist of questionnaires, interviews, computer tasks and video- recordings with both parents. Data collection will start in September 2011 till June 2013.

### Sample size, power and statistical analysis

#### Sample size and power

A randomised controlled trial will be conducted to evaluate the effectiveness of VIG for parents with premature infants. In comparison with the control group (receiving CAU) it is expected that parents in the intervention group (receiving CAU and VIG) exhibit an enhanced quality in parent-infant interactions and they experience less bonding problems. Bonding problems will be measured with the Postpartum Bonding Questionnaire (PBQ) [67] and the quality of the parent-infant interaction will be measured with the EAS [72].

The sample size calculation is based on the identification of a minimal relevant clinical difference on the EAS subscales of 1.15-1.55 points. Considering a dropout rate of 20-30%, a standard deviation of 1.54 points, power of 80% and a significance level of 5%, an average sample size of 23 (17–29) in each group will be sufficient to detect a clinical relevant difference on the EAS subscales This number has been increased to 35 per group, to allow for the anticipated drop out during the first year.

### Data analysis

Multilevel modelling will be performed [95]; a flexible regression technique particularly suited for complex longitudinal datasets. To detect clinical significant differences and considering the maximum number of available pre-term infants, the inclusion of 70 infants per group is deemed sufficient. With approximately 100 (high-risk preterm) births a year in the separate specialised hospitals and approximately 150 low-risk premature and 2400 term births in the general hospitals per year, inclusion of 3 x 70 infants and their parents is feasible.

### Ethical considerations

This study is funded by a Dutch foundation: “Achmea, Foundation Victim and Society”. Ethical and local feasibility approval was obtained for all eight participating hospitals. (Project number: NL24021.060.08, MEC approval: Catharina Hospital, Eindhoven, date: 21-10-2008). All parents will be provided with both verbal and written information about the study and written informed consent is obtained prior to enrolment in the trial.

A data supervising committee will monitor the progress of the study and the inclusion of the parents. No formal stopping rules are in place however funding will only be extended in case of sufficient participating families in the trial.

## Discussion

This is the first prospective study to empirically evaluate the effect of VIG in families with premature infants. The results of this study are relevant for policy, practice and theory for several reasons. Firstly, prematurity constitutes a serious risk factor for adverse parenting. As already mentioned, there are a considerable number of premature births in the Netherlands and a surprisingly high number of infants at risk for child maltreatment. This has become a matter of serious concern for health care policy in general and parents and health care providers in particular. By prospectively examining the process of bonding, this study may contribute to the early detection of bonding problems and adverse parenting. By the use of an easily to administer screening instrument, early bonding problems can be detected and interventions might be suggested. Secondly, the results may also contribute to the implementation of a non-intrusive short-term intervention program for infants at risk for adverse parent-infant interactions (*i.e.*, pre- term infants and infants with pre-peri- and postnatal risk-factors). Family recruitment is expected to be completed in December 2011. First results should be available by 2012.

## Abbreviations

VIG, Video interaction guidance; CAU, Care as usual; GA, Gestational age.

## Competing interests

The authors declare that they have no competing interests.

## Authors’ contributions

The study protocol was developed by HvB, in collaboration with FWW, ME and AV at the department of Developmental Psychology, Tilburg University, Tilburg, the Netherlands and the Association Interactionguidance and Treatment, Foundation and Combination Youth care, Eindhoven, the Netherlands. AT and HH were appointed as PhD-students in 2009 and executed the study until children were 6 months old. RH was appointed to the project as a PhD-student in a later phase and will execute the follow-up study until children are 24 months old. All collaborators are considered as co-authors as they have significantly contributed in the development of the study, obtaining the data, and writing the manuscript. All authors read and approved the final manuscript.

## Pre-publication history

The pre-publication history for this paper can be accessed here:

http://www.biomedcentral.com/1471-2431/12/76/prepub
